# Proteotranscriptomic analyses reveal distinct interferon-beta signaling pathways and therapeutic targets in choroidal neovascularization

**DOI:** 10.3389/fimmu.2023.1163739

**Published:** 2023-03-21

**Authors:** Yuxiang Hu, Siyi Qi, Hong Zhuang, Qiao Zhuo, Yu Liang, Hongyu Kong, Chen Zhao, Shujie Zhang

**Affiliations:** Eye Institute and Department of Ophthalmology, Eye & ENT Hospital, Fudan University, Shanghai, China

**Keywords:** immunity, inflammation, choroidal neovascularization (CNV), interferon-β (IFN-β), proteotranscriptomics

## Abstract

**Aim:**

To investigate the molecular mechanism underlying the onset of choroidal neovascularization (CNV).

**Methods:**

Integrated transcriptomic and proteomic analyses of retinas in mice with laser-induced CNV were performed using RNA sequencing and tandem mass tag. In addition, the laser-treated mice received systemic interferon-β (IFN-β) therapy. Measurements of CNV lesions were acquired by the confocal analysis of stained choroidal flat mounts. The proportions of T helper 17 (Th17) cells were determined by flow cytometric analysis.

**Results:**

A total of differentially expressed 186 genes (120 up-regulated and 66 down-regulated) and 104 proteins (73 up-regulated and 31 down-regulated) were identified. The gene ontology and KEGG pathway analyses indicated that CNV was mainly associated with immune and inflammatory responses, such as cellular response to IFN-β and Th17 cell differentiation. Moreover, the key nodes of the protein–protein interaction network mainly involved up-regulated proteins, including alpha A crystallin and fibroblast growth factor 2, and were verified by Western blotting. To confirm the changes in gene expression, real-time quantitative PCR was performed. Furthermore, levels of IFN-β in both the retina and plasma, as measured by enzyme-linked immunosorbent assay (ELISA), were significantly lower in the CNV group than in the control group. IFN-β treatment significantly reduced CNV lesion size and promoted the proliferation of Th17 cells in laser-treated mice.

**Conclusions:**

This study demonstrates that the occurrence of CNV might be associated with the dysfunction of immune and inflammatory processes and that IFN-β could serve as a potential therapeutic target.

## Introduction

Choroidal neovascularization (CNV), as a pathological process, generally extends along the choroid to the underside of the retinal pigment epithelium (RPE), causing hemorrhage and exudation (1). A variety of pathological conditions can lead to CNV, especially neovascular age-related macular degeneration (AMD) (2). In developed countries, CNV secondary to AMD is the almost universal cause of devastating central visual function damage in the elderly (3). It is predicted that approximately 288 million people will have AMD by 2040, up from 196 million in 2020. In addition, by that time, it is expected that Asia will be the region with the largest number of patients with AMD (4, 5). CNV is a characteristic pathological change in wet AMD; it causes exudation, bleeding, and scars in the macular zone of AMD patients, leading to a decrease in central strength and subsequent loss of vision (6). Although patients with CNV secondary to wet AMD constitute only 20% of all patients with AMD, > 90% of these patients exhibit severe visual impairment (7). AMD-related visual impairment and blindness have become worldwide public health problems that aggravate the already heavy healthcare-related financial burden on society.

The precise etiopathogenesis of CNV is still unknown, but it is generally presumed to result from imbalances in local angiogenesis stimulators and inhibitors that are the result of aging, oxidation, inflammation, and damage to Bruch’s membrane (8). Vascular endothelial growth factor (VEGF) can specifically and directly act on vascular endothelial cells; it is a critical factor in promoting angiogenesis, particularly during the onset of CNV (9). In recent years, anti-VEGF drugs have been extensively used in clinics as first-line drugs for the treatment of wet AMD (10). Such drugs can reduce blood leakage from new blood vessels, reduce neovascularization, and promote foveal edema regression. However, although these drugs can help to partially restore vision and delay disease progression, they cannot cure wet AMD (6). Thus, there is a considerable need to clarify the mechanisms underlying the onset of secondary CNV during the course of AMD and, in turn, to identify potential targets for the treatment of AMD.

Previous studies have demonstrated that the onset and progression of CNV are mediated by various regulatory factors (11, 12). However, many molecular components of the pathophysiology of CNV remain unknown, especially in the adult population. Proteomic analysis is considered one of the most advanced exploratory research methods that can be used to discover new protein biomarkers of clinical significance (13). Multiple studies have employed proteomic analysis to study AMD (14). Furthermore, RNA transcriptome analysis of human AMD in donor eyes revealed that multiple pathogenic pathways (e.g., angiogenesis, extracellular matrix remodeling, inflammation, and immune responses) were perturbed in retinal pigment epithelium (RPE) cells (15, 16). Although proteomics and transcriptomics have improved general knowledge regarding CNV, more systematic studies of the relationship between proteomes and transcriptomes may reveal novel molecular alterations in CNV biology.

In the present research, we constructed a laser-induced CNV mouse model and then combined proteomic and transcriptomic (i.e., proteotranscriptome) analyses to identify the core genes, biological processes, and signaling pathways involved in the onset of CNV. In addition, we focused on the functions of interferon-β (IFN-β) and related potential therapy for CNV.

## Materials and methods

### Animals

The experimental subjects were approximately 8-week-old male C57BL/6J mice, purchased from Gempharmatech Company. These mice were raised in the animal house of Fudan University at a constant temperature (23 ± 1°C) and humidity (40%–60%), with a light/dark cycle of 12 h, and freely available water and standard feed. At the end of the experiment, they were sacrificed by cervical dissection. The animal experiment operation procedures in the research were approved by the Animal Ethics Committee of Fudan University.

### Laser-induced CNV model

Before the operation, the mice were intraperitoneally injected with 1.25% tribromoethanol (0.2 mL/10 g) for anesthesia. Subsequently, 0.4% oxybuprocaine hydrochloride was used for corneal anesthesia, and tropicamide was used for pupil dilation. The cornea was lubricated with carbomer eye gel to prevent drying. Three laser spots were distributed evenly around the optic nerve of each eye utilizing a fundus laser system (Zeiss, Germany) with an energy setting of 100 mW and a duration of 100 ms.

### Fundus photography and immunohistochemistry

Seven days after the induction of CNV, the mice were anesthetized, and their pupils dilated and lubricated as described above. Thereafter, the lens of the fundus imaging system was placed in contact with the eye to be tested, and the focal length was adjusted as necessary for image clarity; photos were then acquired. After fundus photography, the mice remained anesthetized and were sacrificed by cervical dislocation. Each eye was then enucleated and stabilized in 4% paraformaldehyde at room temperature for 1 hour. Under a stereomicroscope, the eye was circumferentially dissected along the corneoscleral limbus; the anterior segment components (e.g., cornea, iris, and lens) were eliminated and the neural retina was detached, thus yielding RPE/choroidal flat mounts. Each RPE/choroidal plane flat mount was radially cut into a petal shape centered on the optic disk. Thereafter, they were incubated with anti-plant lectin B4 antibody (1:100 dilution, Sigma, USA) overnight at 4°C after being permeabilized and blocked at room temperature for 1 hour. Subsequently, a secondary antibody (1:500 dilution, Sigma, USA) was incubated for 1 hour at room temperature in dark on each flat mount. For observation, the flat mount was placed on glass slides and inlaid in a fluorescence mounting medium.

### RNA sequencing and data analysis

Total RNA was extracted from each retinal sample using RNeasy Kits (Qiagen, Germany), and its purity and concentration were evaluated. Thereafter, a cDNA library was constructed using a commercial reverse transcription kit (Illumina, USA). The sequencing of these libraries was performed on the HiSeq platforms (Illumina, USA). The results indicated the locations of the reads within the reference genome, along with information regarding sequence features specific to the sequenced sample. The number of counts for each sample gene was normalized (BaseMean values were used to estimate expression levels); fold change (FC) was calculated, and a negative binomial distribution was used to verify the significance of differences in reads. Differentially expressed genes (DEGs) were categorized according to FC and read numbers. Unsupervised hierarchical clustering demonstrated a clear separation between the two groups; up-regulation and down-regulation trends were consistent, reflecting obvious differences between the two groups of genes. Furthermore, The Gene Ontology (GO) and the Kyoto Encyclopedia of Genes and Genomes (KEGG) databases were analyzed to identify the DEGs, as follows: the species genes were used as the background list, while the differential gene list was used as the candidate list for screening relative to the background list. *p*-values were computed and corrected by the hypergeometric distribution test. The screening criteria for DEGs were FC ≥ 1.5 and *p* < 0.05. The RNAseq datasets that are presented in the study were deposited in the Genome Sequence Archive repository, accession number CRA009762.

### Mass spectrometry-based proteomic analysis

Total protein was extracted from each retinal sample. After trypsinization and labeling, the remaining portions of each extract were mixed and chromatographically separated by weight. The sample was loaded onto a chromatographic column (C18, 100 µm × 2 cm), and then separated by a reversed-phase liquid chromatographic column (C1_8_, 75 µm × 15 cm) (both Thermo Fisher, USA). Later, tandem mass spectrometry (MS/MS) was conducted to analyze the separated samples using a HF-X mass spectrometer (Thermo Fisher, USA). A collision energy of 35 eV was applied for all MS/MS spectra acquired using data-dependent high-energy collisional fragmentation. Putative proteins were distinguished by comparing the MS outcome with the UniProt database after using the Sequest HT score > 0 and unique peptide ≥ 1. Significant differences between samples were defined as FC ≥ 1.2 and *p* < 0.05 for these putative proteins. GO and the KEGG analysis of differentially expressed proteins (DEPs) was conducted in accordance with the process described above. In addition, the STRING database was employed to analyze DEPs to obtain protein–protein interaction (PPI) networks, and the results were visualized using Cytoscape software. Confidence scores of > 0.7 were considered statistically significant. The proteomic datasets were deposited in the ProteomeXchange repository, accession number PXD039971.

### Quantitative real-time PCR

RNA was extracted in accordance with the process mentioned above and then reverse transcribed to cDNA using EZscript RT Mix (EZBioscience, USA) and random primers. Quantitative real-time PCR (qPCR) was carried out by the LightCycler 96 qPCR system (Roche, Switzerland) using the SYBR Green qPCR mix kit (EZBioscience, USA). The sequences of primer pairs were as follows: Prok1: 5′-GCCTGTGAACGAGATATCCAGTTGTG-3′ and 5′-CTGGGTGGCACTCCTCTCCTTC-3′; Adcy1: 5′-TCACCCAGCCTAAGACGGATCAC-3′ and 5′-TCAGTAGCCTCAGCCACGGATG-3′; Col4a3: 5′-ACCAGGACCACCAGGACCAAAG-3′ and 5′-GTGACCAGGACTCCCAGGACTC-3′; Col4a4: 5′-CCAAGAGGTGATGCGGGAGATTTC-3′ and 5′-CCAGTAAGCCATTCAGACCAGGAG-3′; β-Actin: 5′-TATGCTCTCCCTCACGCCATCC-3′and 5’-GTCACGCACGATTTCCCTCTCAG-3′. The data were analyzed using the 2^−ΔΔCT^ method.

### Western blotting

Sample proteins were extracted and quantified as previously described, and boiled with the loading buffer (Beyotime, China) for 5 min. Subsequently, they were separated using pre-cast gels in SDS-PAGE electrophoresis buffer (Beyotime, China) at 130 V for 90 min, and then transferred to polyvinylidene fluoride membranes at 350 mA for 40 min. After being blocked in 5% non-fat milk, the membranes were incubated with the primary antibodies, anti-alpha A crystallin (CRYAA, Abcam, USA) and anti-fibroblast growth factor 2 (FGF2, Abcam, USA), at 4°C overnight, and with goat anti-rabbit IgG (Thermo Fisher, USA) secondary antibody for 1 hour. Blots were developed using a gel imaging system (Image Quant 350, GE Healthcare, USA). Densitometric analysis was executed using ImageJ software.

### Enzyme-linked immunosorbent assay

Mercantile enzyme-linked immunosorbent assay (ELISA) kits (Multisciences Biotech, China) were applied to determine the IFN-β levels in the plasma and retina of wild-type (WT) and CNV mice. The experimental procedure was implemented in strict accordance with the product instructions.

### IFN-β treatment

The mice in the treatment group received intraperitoneal injections of 10,000 IU of recombinant human IFN-β1a (PeproTech, USA) every other day until the end of the experiment (17). In the control group, intraperitoneal injections of phosphate-buffered saline (PBS) were used. All treatment manipulations were performed by the same research staff. Mice in the experimental and control groups were mixed and housed in randomly assigned cages after they had been labeled.

### Flow cytometry

On day 7, retinal neuroepithelium specimens were collected from the mice. The specimens were ground and lysed with erythrocyte lysis buffer to produce single-cell suspensions. The suspensions were centrifuged at 1,200 rpm for 5 min; the resulting cells were resuspended in PBS. The cells were then stained with CD4 at room temperature for 15 minutes and with interleukin 17A (IL-17A) at 4°C for 30 min after fixation and permeabilization, and finally analyzed by flow cytometry. The outcomes were evaluated using FlowJo software.

### Statistical analysis

GraphPad Prism 9.0 (GraphPad, USA) and SPSS 22.0 (IBM Corp., USA) were used to conduct statistical analysis. Student’s *t*-test was used to make comparisons between the two groups. *p*-values of ≤ 0.05 were considered significant.

## Results

### Transcripts altered and biological pathways enriched in CNV retina

**Figure 1:**
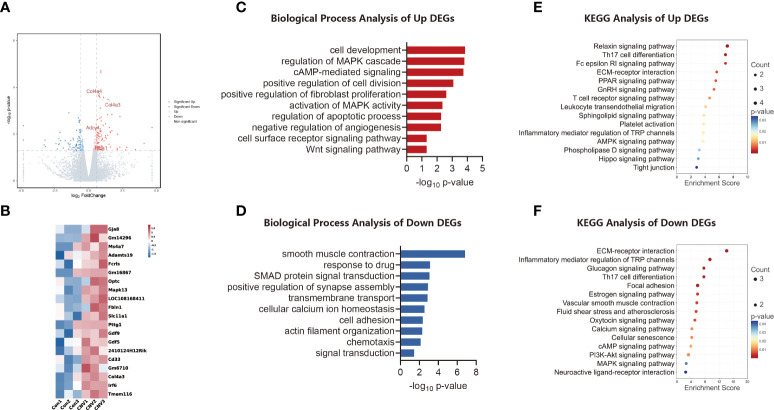
Transcriptomic analysis of the CNV mouse retina. **(A)** Volcano plot visualizing transcriptomic alterations, where red dots represent significantly upregulated genes, green dots represent significantly downregulated genes, and some red dots are labeled to illustrate high regulation. **(B)** Heatmap of the top 20 differentially expressed genes (DEGs) between CNV and control retinas. **(C)** Bar chart of sectional biology progress (BP) gene ontology (GO) terms of upregulated DEGs. **(D)** Bar chart of sectional biology progress (BP) gene ontology (GO) terms of downregulated DEGs. **(E)** Bubble plot of partial enriched Kyoto Encyclopedia of Genes and Genomes (KEGG) pathway terms of upregulated DEGs. **(F)** Bubble plot of partial enriched KEGG pathway terms of downregulated DEGs.

The data were stratified according to group, and the corresponding net values were screened for differences. A total of 186 genes with significant differences were obtained: 120 were up-regulated and 66 were down-regulated. As shown in **Figure 1A**, various genes were expressed differently between the groups. To visualize the expression profile of genes in each sample and the differences between groups, the top 20 altered genes were plotted in heatmap format (**Figure 1B**).

Biological process (BP) GO annotation revealed the functional items of the identified DEGs. Trends in enrichment in up-regulated and down-regulated gene expression of DEGs are depicted in **Figures 1C, D**, respectively. DEGs that showed significantly up-regulated expression were those involved in cell development and regulation of the MAPK cascade, whereas DEGs whose expression was significantly down-regulated were those involved in smooth muscle contraction and chemotaxis.

The KEGG database was analyzed to identify DEGs in retinal signaling pathways. The main pathways involved in the up-regulation of genes in the CNV group included the relaxin signaling pathway and Th17 cell differentiation, as shown in **Figure 1E**. The main pathways involved in down-regulated genes in the CNV group included extracellular matrix (ECM)–receptor interaction and Th17 cell differentiation, as shown in **Figure 1F**.

### Proteins altered and biological pathways enriched in CNV retina

**Figure 2:**
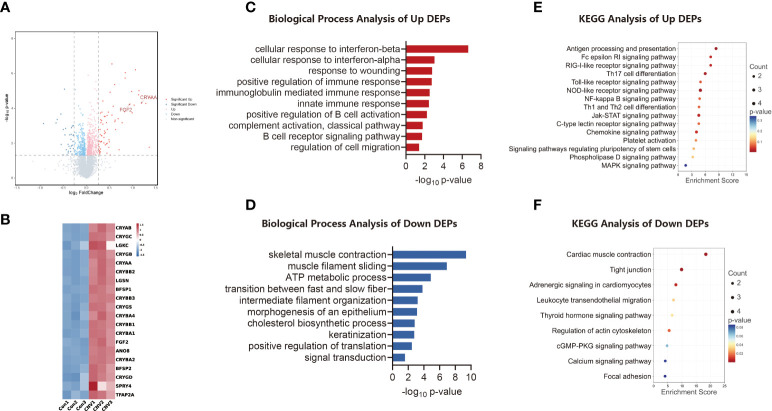
Proteomic analysis of CNV mouse retina. **(A)** Volcano plot visualizing proteomic alterations, where red dots represent proteins upregulated in the CNV group (compared to the control group; 73 proteins), blue dots represent proteins downregulated in the CNV group (31 proteins). **(B)** Heatmap of the top 20 differentially expressed proteins (DEPs) between CNV and control retinas. **(C)** Bar chart of sectional biology progress (BP) gene ontology (GO) terms of upregulated DEPs. **(D)** Bar chart of sectional biology progress (BP) gene ontology (GO) terms of downregulated DEPs. **(E)** Bubble plot of partial enriched Kyoto Encyclopedia of Genes and Genomes (KEGG) pathway terms of upregulated DEPs. **(F)** Bubble plot of partial enriched KEGG pathway terms of downregulated DEPs.

A volcano plot analysis (**Figure 2A**) revealed that there were 104 DEPs. Heatmap analysis (**Figure 2B**) showed the top 20 variant proteins, including CRYAA and FGF2.

Different expression patterns for proteins were analyzed using GO enrichment analysis. **Figures 2C, D** shows the trends of BP enrichment in up-regulated and down-regulated DEPs, respectively. Significantly up-regulated proteins included those involved in the cellular response to interferon-beta and in the innate immune response, while significantly down-regulated proteins included those involved in muscle filament sliding and signal transduction.

The differential proteins identified were retrieved and enriched through the KEGG signaling pathway database. A bubble plot was then constructed to illustrate the consequence of enrichment (*p*-value), the amount of enrichment, and the enrichment index. As indicated in **Figure 2E**, the up-regulated proteins were chiefly involved in signaling pathways, including those for antigen processing and presentation and Th17 cell differentiation. As shown in **Figure 2F**, the down-regulated proteins were principally those involved in processes such as cardiac muscle contraction and the function of tight junctions.

### Integration of the transcriptomics and proteomics

**Figure 3:**
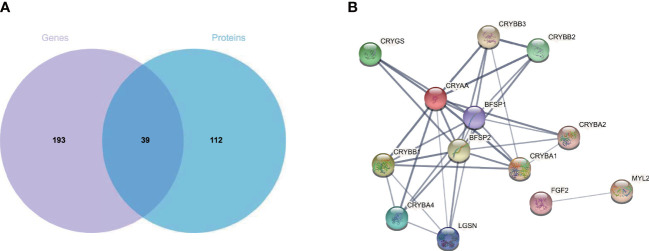
Combined transcriptome and proteome analysis. **(A)** Venn diagram of mRNA and proteins from CNV and control retinas. **(B)** Protein–protein interaction analysis of differentially expressed proteins. CNV, choroidal neovascularization; mRNA, messenger RNA.

Matching analysis of the identified genes and proteins identified 39 overlapping targets (**Figure 3A**), 16 of which were co-up-regulated and six of which were co-down-regulated (**Table 1**). In addition, the biological processes in the interaction networks covered 13 proteins (**Figure 3B**). Key nodes in PPI networks mainly involved up-regulated proteins such as CRYAA and FGF2.

**Table 1:** Characteristics of the 39 overlapping targets in identified genes and proteins.

Gene name	Description	Genes		Proteins	
		Fold change	Regulation	Fold change	Regulation
*Ngp*	Neutrophilic granule protein	0.00	DOWN	0.76	DOWN
*Cd5l*	CD5 antigen-like	0.00	DOWN	1.20	UP
*Orm1*	Alpha-1-acid glycoprotein 1	0.00	DOWN	1.37	UP
*Cryge*	Gamma-crystallin E	0.08	DOWN	1.33	UP
*Aox3*	Aldehyde oxidase 3	0.20	DOWN	0.82	DOWN
*Gbp4*	Guanylate-binding protein 4	0.48	DOWN	1.21	UP
*S100a4*	Protein S100-A4	0.50	DOWN	1.23	UP
*Myl2*	Myosin regulatory light chain 2, ventricular/cardiac muscle isoform	0.54	DOWN	0.53	DOWN
*Mt2*	Metallothionein-2	0.55	DOWN	1.54	UP
*S100a6*	Protein S100-A6	0.60	DOWN	1.20	UP
*Mt1*	Metallothionein-1	0.60	DOWN	1.26	UP
*Hp*	Haptoglobin	0.60	DOWN	1.23	UP
*Spry4*	Protein sprouty homolog 4	0.61	DOWN	1.63	UP
*Gfap*	Glial fibrillary acidic protein	0.62	DOWN	1.47	UP
*Itga2*	Integrin alpha-2	0.62	DOWN	0.71	DOWN
*Nccrp1*	F-box only protein 50	0.63	DOWN	0.83	DOWN
*Jsrp1*	Junctional sarcoplasmic reticulum protein 1	0.64	DOWN	0.81	DOWN
*Cryga*	Gamma-crystallin A	0.64	DOWN	1.32	UP
*Mt3*	Metallothionein-3	0.66	DOWN	1.31	UP
*Crybb3*	Beta-crystallin B3	1.62	UP	2.09	UP
*Plbd1*	Phospholipase B-like 1	1.67	UP	1.22	UP
*Rac2*	Ras-related C3 botulinum toxin substrate 2	1.90	UP	1.36	UP
*Fgf2*	Fibroblast growth factor 2	1.93	UP	1.91	UP
*Crybb1*	Beta-crystallin B1	1.95	UP	1.97	UP
*Steap4*	Metalloreductase STEAP4	1.97	UP	1.41	UP
*Apom*	Apolipoprotein M	2.13	UP	1.40	UP
*Bfsp2*	Phakinin	2.27	UP	1.78	UP
*Cryba1*	Beta-crystallin A1	2.52	UP	1.92	UP
*Lgsn*	Lengsin	2.67	UP	2.20	UP
*Crygs*	Gamma-crystallin S	2.68	UP	2.05	UP
*Cryaa*	Alpha-crystallin A chain	2.92	UP	2.30	UP
*Cryba4*	Beta-crystallin A4	2.96	UP	1.99	UP
*Bfsp1*	Filensin	2.99	UP	2.19	UP
*Myh3*	Myosin-3	3.29	UP	0.65	DOWN
*Crybb2*	Beta-crystallin B2	3.33	UP	2.29	UP
*Cryba2*	Beta-crystallin A2	3.54	UP	1.79	UP
*Krt17*	Keratin, type I cytoskeletal 17	Inf	UP	0.68	DOWN
*Myh8*	Myosin-8	Inf	UP	0.71	DOWN
*Krt76*	Keratin, type II cytoskeletal 2 oral	Inf	UP	0.80	DOWN

### Validation of selected genes and proteins altered in CNV retina

**Figure 4:**
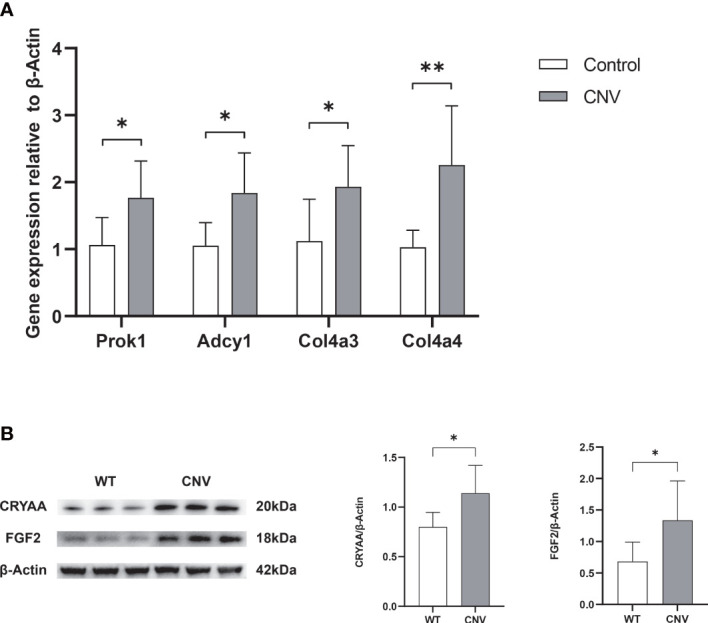
Verification of selected genes and proteins. **(A)** RNA expression of Prok1, Adcy1, Col4a3, and Col4a4 was measured in retinas using qPCR. *n* = 6; mean ± SD; Student’s *t*-test; *p < 0.05, **p < 0.01. **(B)** The content of Cyraa and FGF2 proteins in retinas was detected by Western blot. *n* = 6; mean ± SD; Student’s *t*-test; *p < 0.05, **p < 0.01. qPCR, quantitative real-time PCR.

To validate the screening results, we extracted total RNA from the retinas of mice in both the CNV group and the control group, and then reverse transcribed this RNA into cDNA. From the screening results, we selected four genes (*Prok1*, *Adcy1*, *Col4a3*, and *Col4a4*) that may be involved in neovascularization to serve as qPCR target genes. β-Actin served as the reference gene. As illustrated in **Figure 4A**, the levels of *Prok1*, *Adcy1*, *Col4a3*, and *Col4a4* were significantly increased in the retinas of mice with CNV (*p* < 0.05 in all cases), consistent with the results of the transcriptomic analysis. To validate the MS results, we conducted a semi-quantitative analysis and exploration of some differential protein molecules (i.e., CRYAA and FGF2) by Western blotting. As shown in **Figure 4B**, both CRYAA and FGF2 levels were strikingly up-regulated in the CNV group compared with the control group (*p* < 0.05 in both cases), consistent with the results of proteomic analysis.

### Enrichment of IFN-β in CNV retina

**Figure 5:**
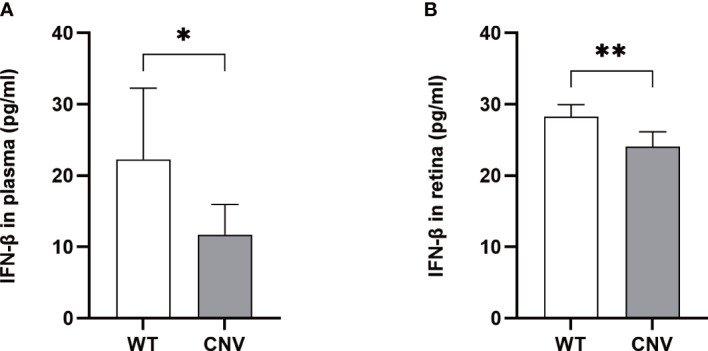
Level of IFN-β in the CNV mice. The levels of IFN-β were significantly lower in the plasma **(A)** and retina **(B)** of CNV mice compared with the WT cohort. *n* = 6; mean ± SD; Student’s *t*-test; *p < 0.05, **p < 0.01. CNV, choroidal neovascularization.

To determine if the expression of IFN-β contributes to the progression of CNV, we analyzed IFN-β in plasma and retina samples from mice with CNV and control mice by ELISA. As shown in **Figure 5**, serum and retinal IFN-β levels were dramatically down-regulated in the CNV group compared with the control group (*p* < 0.05 in both cases).

### IFN-β therapy stimulates Th17 proliferation and limits CNV

**Figure 6:**
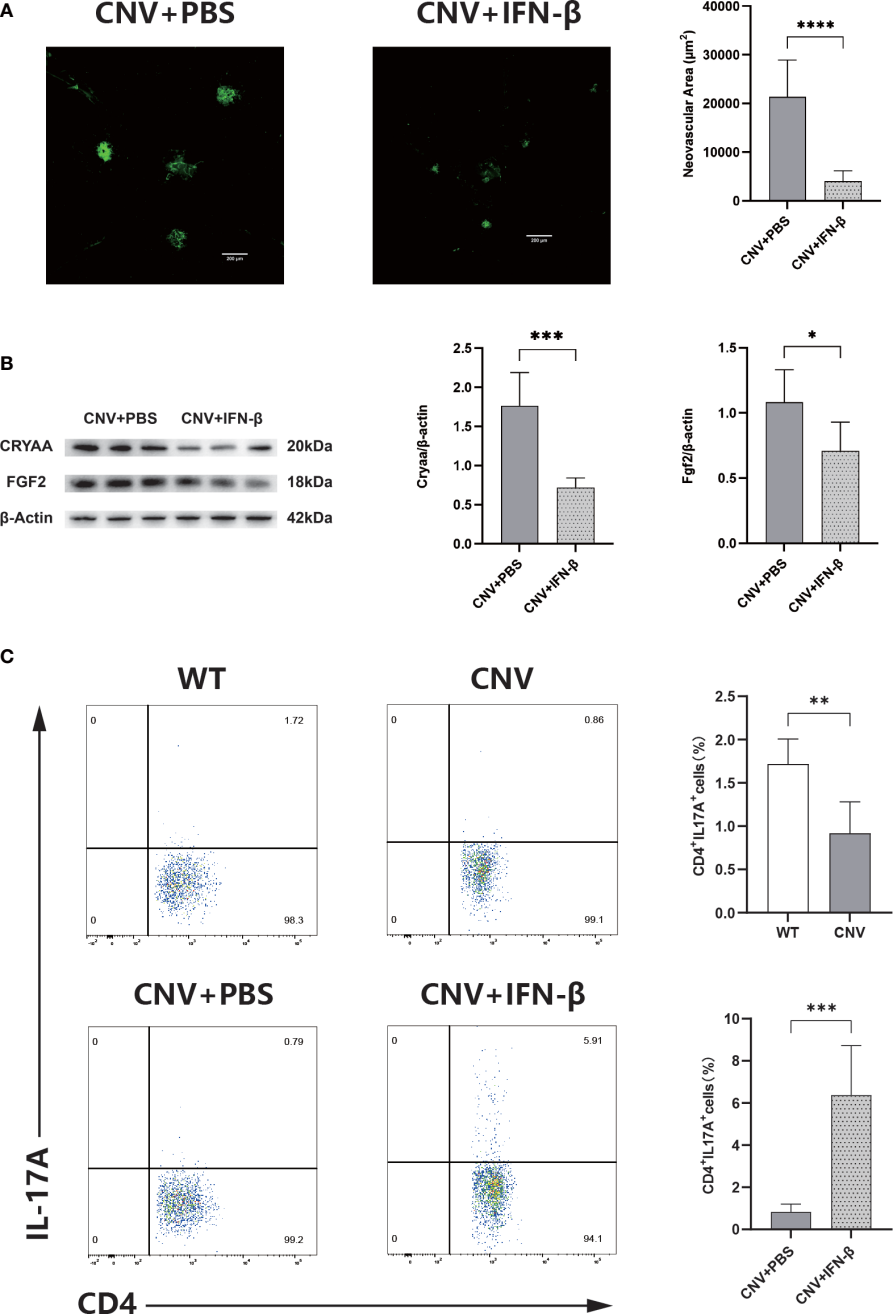
IFN-β therapy limits CNV. **(A)** Representative images of IB4-stained choroidal flat mounts 7 days after laser coagulation in the PBS-treated (control) and IFN-β-treated groups. *n* = 6; mean ± SEM; Student’s *t*-test; ****p < 0.0001. **(B)** Western blot analysis of the expression levels of Cyraa and FGF2 in retinas in the PBS-treated and IFN-β-treated groups. *n* = 6; mean ± SD; Student’s *t*-test; *p < 0.05. **(C)** Representative flow charts of the proportion of Th17 cells from the retinas of wild-type (WT) and CNV groups, and the proportion of Th17 cells from the retinas of the PBS-treated and IFN-β-treated groups. *n* = 6; mean ± SD; Student’s *t*-test; **p < 0.01, ***p < 0.001. CNV, choroidal neovascularization; IFN-β, interferon-β; PBS, phosphate-buffered saline.

The IFN-β-treated group had significantly smaller CNV lesions than the PBS-treated control group (*p* < 0.05; **Figure 6A**) and substantially decreased expression levels of CRYAA and FGF2 (*p* < 0.05 in both cases; **Figure 6B**) on day 7 after laser photocoagulation. In addition, flow cytometry analysis was used to assess the activity expression of Th17 cells in the retina of CNV mice. The proportion of Th17 cells was much lower in the CNV group than in the control group; in the IFN-β-treated group, the proportion was noticeably increased compared with the PBS-treated control group (*p* < 0.05 in both cases; **Figure 6C**).

## Discussion

The onset of CNV involves many complex mechanisms and pathways, which have not been fully elucidated. To characterize CNV pathogenesis, we constructed a mouse model of laser-induced CNV, then conducted combined proteomic and transcriptomic analyses. In all, we found that 186 genes and 104 proteins were dysregulated in the retinas of mice with CNV compared with control mice. These altered genes and proteins are involved in numerous pathways, including inflammation, metabolism, and immune regulation. In addition, we identified 21 proteins that were consistent with DEGs, of which 16 were up-regulated and five were down-regulated. Furthermore, PPI network analysis revealed that CRYAA and FGF2 have important roles in CNV pathogenesis.

Usefully, genetic studies in patients with AMD have discovered multiple susceptibility loci for AMD; most of the genetic risk is shared between AMD and CNV (18). In this study, the levels of *Col4a3* and *Col4a4* were up-regulated in mice with CNV compared with mice in the control group. Using RNAseq analysis, Fletcher et al. found that the levels of many members of the collagen family (e.g., col4a1 and col4a2) were noticeably up-regulated in the RPE/choroid of patients with CNV (19). This divergence in test results may be related to the different tissue sources. Yu et al. found a protective locus for AMD in Col4a3 in a meta-analysis of European patients (20). Another meta-analysis indicated that Col4a3 was significantly connected with polypoidal choroidal vasculopathy, a subtype of neovascular AMD particularly prevalent in East Asians (21). These investigations demonstrate that *Col4a3* may play an important role in AMD pathogenesis. Although *Col4a4* is widely regarded as a pathogenic factor in diseases such as Alport syndrome (progressive glomerulonephritis, lens defects, and hearing loss) (22), it has rarely been regarded as a contributing factor in AMD or CNV. Additionally, Prok1 is an angiogenic growth factor with vital roles in regulating the growth of organ blood vessels and tumor blood vessels (23). In these studies, *Prok1* levels were dramatically higher in the CNV group than in the control group. To our knowledge, this is the first report of such a finding in a CNV model. Because our result is consistent with previous findings (24, 25), we presume that *Prok1* has a vital role in the onset of CNV. In addition, as a member of the ADCY superfamily, *Adcy1* is thought to be involved in tumor angiogenesis (26). In our study, *Adcy1* levels were radically up-regulated in the CNV group compared with the control group. This finding is presumably the first report of such a difference in a CNV model; it suggests that *Adcy1* is involved in CNV formation. On the other hand, we reviewed other transcriptomic studies on CNV and AMD and found that the results of our study did not identify the presence of common genes related to neovascular diseases, such as *PNPLA2*, *MFGE8*, and *DDIT4* (27). This may be because of the restricted sample numbers used in our study. In future studies, we will analyze more samples to verify the conclusions of this study.

The GO and KEGG pathways were analyzed for the DEGs identified in this study. The results indicated that many abnormal BP and signaling pathways (e.g., the synthesis of ECM structural components, the function of celll junctions, actin filament binding, the relaxin signaling pathway, and the PI3K-Akt pathway) are involved in the pathological progression of CNV. These processes and components also participate in normal cell proliferation and migration during angiogenesis. Moreover, the results of the GO analysis of the DEGs were similar to the proteomic analysis of differential protein enrichment, indicating that the gene regulatory trends were consistent with protein regulatory trends. The ECM is an essential component of the vascular microenvironment, and directly or indirectly regulates all essential cellular functions critical for angiogenesis (e.g., cell adhesion, migration, proliferation, differentiation, and lumen formation) (28). Accordingly, the onset of CNV is inseparable from the synthesis (or function) of the structural components of the ECM. Relaxin is an insulin-like polypeptide hormone; most studies have focused on its role in the regulation of angiogenesis during pregnancy, but there have been a few reports of its association with CNV (29). Considering that relaxin can specifically induce VEGF expression to regulate angiogenesis (30), it also has been suggested to play a role in the development of CNV, and the results of this study support this viewpoint. The PI3K-Akt pathway plays pivotal roles in intracellular signaling, which regulates cell proliferation and motility (31). The pathogenesis of CNV involves the activation of the PI3K-Akt pathway, which causes lactic acid fermentation (i.e., the Warburg effect) and induces VEGF expression, in turn leading to the development of CNV (32). In addition, it has been shown that repressing the PI3K-Akt pathway in the choroid by various means can effectively block the onset of CNV (33, 34). Therefore, the identification of suitable PI3K-Akt pathway inhibitors may be a means of counteracting the onset and development of CNV in wet AMD patients. Furthermore, in contrast to the proteomics findings, the KEGG pathway analysis of transcriptome data showed greater involvement of immune factors, including changes in pathways related to immune inflammation (e.g., Th17 cell differentiation). Therefore, we speculate that the variations in mRNA expression cause changes in the biological functions of cells targeted by immunity-related inflammation. Thus, an immune mechanism may participate in the onset and progression of CNV.

In this study, GO analysis of DEPs was implemented to explore the effects on biological functions. The DEPs had significant effects of CNV on molecular functions such as eye structure development, cellular structure and function, immune activity, ATP metabolism, and signal transduction activity. Furthermore, KEGG pathway analysis revealed significant changes in NLR signaling, Th17 cell differentiation, and tight junction regulation. These enriched biological functions and pathways were also identified in the enrichment analysis of DEGs. Notably, the choriocapillary diameter, blood flow, and oxygen tension are radically increased in the macula compared with the peripheral retina, although pericyte coverage is lower. Choroidal vessels in the macula are more susceptible to pathological changes under stress (35). In addition, the outer third of the retina remains physiologically completely avascular; it relies on the choroidal system to receive essential nutrients and oxygen. When the choroidal vasculature is damaged, this supply chain is disrupted and the onset of CNV begins (36). RPE dysfunction can also lead to disruptions in the supply chain. Critical factors involved in RPE dysfunction include age-dependent changes in phagocytosis and metabolism in postmitotic RPE cells (37). Previous studies have confirmed that differences in retinal tissue structure and cell function have a vital impact on the occurrence of CNV. The retina maintains its normal function through metabolism, which provides energy to the retina; metabolites have important roles in maintaining retinal homeostasis (38). Metabolic alterations (e.g., abnormal ATP metabolism) are presumed to result from combinations of genetic and environmental factors; abnormal cellular metabolism is therefore firmly related to the onset of disease, especially in multifactorial disorders such as AMD (39). The NLR pathway is known to regulate the formation of inflammasomes and stimulate the production of both IL-1β and IL-18, thereby participating in the inflammatory response (40). Furthermore, the NLR pathway has been shown to promote ocular inflammation by activating the production of anti-inflammatory and pro-inflammatory cytokines; such inflammation is closely associated with angiogenesis (41). In AMD, local immunity and inflammatory infiltration promote drusen formation, RPE atrophy, Bruch’s membrane rupture, and CNV onset (42). In addition, inflammatory cytokines can also induce VEGF production, which in turn initiates CNV in AMD; macrophages and lymphocytes are present in the retina during the active phase of CNV (43). It was found that the level of IL-17 was greatly elevated in the eyes of AMD patients and that the inhibition of IL-17 had neuroprotective effects on the eyes of mice with focal retinal degeneration (44). As a characteristic secretory factor of Th17 cells, IL-17 can stimulate the production of VEGF. It also induces cell invasion, migration, and angiogenesis in endothelial cells (45). Based on the previous literature, we suspect that Th17 cells are involved in the onset of CNV.

The hub genes of PPI networks mainly involved proteins that were up-regulated in our analyses, including CRYAA and FGF2. We selected the CRYAA and FGF2 proteins for validation of the results of MS screening; these findings confirmed that the levels of CRYAA and FGF2 in the CNV group were greatly up-regulated compared with the control group. CRYAA, a subunit of α-crystallin, is present in the normal retina; it participates in various retinopathies (46). In addition, knocking out α-crystallin leads to the inhibition of pathological neovascularization through VEGF and VEGFR2 signaling (47). These findings prove that CRYAA may be a valuable target for CNV prevention. FGF2 is an effective factor to stimulate angiogenesis (48). *In vivo* and *in vitro* analyses revealed that FGF2 regulates pathogenic angiogenesis through the activation of STAT3 (49). Because it functions as a key mediator of abnormal neovascularization, FGF2 may be useful in the advance of multi-targeted therapies for blinding eye disorders. The above studies suggest that the production of new blood vessels in patients with CNV may be promoted through alterations to the PPI networks. In this research, the functional analysis of differential proteins illustrated that differential proteins up-regulated in the retina of the CNV group were involved in cell proliferation, migration, and angiogenesis; thus, CNV may be mediated by variations in the expression patterns of these proteins.

Based on analysis of the aforementioned transcriptomic studies, we speculated that CNV was associated with the IFN-β-mediated regulation of the Th17 cell-mediated inflammatory response. In this research, the size of CNV lesions was greatly reduced in CNV mice that received systemic IFN-β treatment compared with mice in the control group. Langmann et al. (17) found that the administration of systemic IFN-β treatment to CNV mice reduced lesion size considerably in the late stage of the disease. Kimura et al. (50) proposed that IFN-β could be able to delay the multiplication of human umbilical vein endothelial cells and enhance the proliferation of RPE cells. Therefore, we speculate that IFN-β can restrain CNV formation. Furthermore, we explored the numbers of Th17 cells among retina cells in CNV mice. In this study, the proportion of Th17 cells among retina cells was considerably lower in CNV mice than in mice in the control group; in contrast, the proportion of Th17 cells was meaningfully increased in the group receiving IFN-β treatment compared with the control group. Moreover, the IL-17 level in peripheral blood and macular cells was significantly increased in AMD patients (44, 51). IL-17 can independently promote CNV formation without the involvement of VEGF (52). IL-17, as a characteristic secretion of Th17 cells, is involved in ocular neovascularization (45). However, the findings of some studies suggest that the elevated level of IL-17 in CNV originates from γδT cells, rather than from Th17 cells (52). This discrepant conclusion may be related to the use of different intervention methods. In addition, IFN-β plays divergent roles in discrete stages of Th17 differentiation (53). Referring to the above information, we speculate that the use of IFN-β to promote the proliferation of Th17 cells can inhibit CNV progression, which cannot be solely achieved by the secretion of IL-17. Thus far, there have been few studies concerning the role that Th17 cells play in inhibiting the progression of CNV. These findings require validation in future studies.

In conclusion, we used transcriptomic and proteomic methods to analyze CNV in this study; the results reflected overall changes in RNA and protein expression. Moreover, we identified a series of biological processes (e.g., inflammation and immune mechanisms) that are involved in CNV pathogenesis, along with the signaling pathways that may lead to CNV pathogenesis. Furthermore, the joint analysis of differential genes and differential proteins led to the identification of CRYAA and FGF2, key proteins involved in CNV; this finding is an important insight that will promote a further understanding of the onset and progression of CNV. In addition, IFN-β can inhibit CNV lesions while increasing immune cell activation. However, a notable shortcoming of this study was its lack of functional appraisal concerning the identified mRNAs and proteins. Consequently, the exact roles and mechanisms of altered mRNAs and proteins in CNV ought to be further studied.

## Data availability statement

The datasets presented in this study can be found in online repositories. The names of the repository/repositories and accession number(s) can be found in the article/supplementary material.

## Ethics statement

The animal study was reviewed and approved by the Animal Ethics Committee of Fudan University.

## Author contributions

YH and SQ performed experiments and manuscript writing. HZ and QZ analyzed the data. YL and HK validated data collection. CZ and SZ revised and finalized the manuscript. All authors commented on and revised the manuscript. All authors contributed to the article and approved the submitted version.
